# Reanalysis datasets outperform other gridded climate products in vegetation change analysis in peripheral conservation areas of Central Asia

**DOI:** 10.1038/s41598-020-79480-y

**Published:** 2020-12-31

**Authors:** Harald Zandler, Thomas Senftl, Kim André Vanselow

**Affiliations:** 1 Working Group of Climatology, Department of Geography, University of Bayreuth, Universitätsstr. 30, 95447 Bayreuth, Germany; 2 Bayreuth Center of Ecology and Environmental Research, University of Bayreuth, Dr. Hans-Frisch-Straße 1-3, 95448 Bayreuth, Germany; 3 Institute of Geography, Friedrich-Alexander-Universität Erlangen-Nürnberg, Wetterkreuz 15, 91058 Erlangen, Germany

**Keywords:** Climate sciences, Ecology, Environmental sciences

## Abstract

Global environmental research requires long-term climate data. Yet, meteorological infrastructure is missing in the vast majority of the world’s protected areas. Therefore, gridded products are frequently used as the only available climate data source in peripheral regions. However, associated evaluations are commonly biased towards well observed areas and consequently, station-based datasets. As evaluations on vegetation monitoring abilities are lacking for regions with poor data availability, we analyzed the potential of several state-of-the-art climate datasets (CHIRPS, CRU, ERA5-Land, GPCC-Monitoring-Product, IMERG-GPM, MERRA-2, MODIS-MOD10A1) for assessing NDVI anomalies (MODIS-MOD13Q1) in two particularly suitable remote conservation areas. We calculated anomalies of 156 climate variables and seasonal periods during 2001–2018, correlated these with vegetation anomalies while taking the multiple comparison problem into consideration, and computed their spatial performance to derive suitable parameters. Our results showed that four datasets (MERRA-2, ERA5-Land, MOD10A1, CRU) were suitable for vegetation analysis in both regions, by showing significant correlations controlled at a false discovery rate < 5% and in more than half of the analyzed areas. Cross-validated variable selection and importance assessment based on the Boruta algorithm indicated high importance of the reanalysis datasets ERA5-Land and MERRA-2 in both areas but higher differences and variability between the regions with all other products. CHIRPS, GPCC and the bias-corrected version of MERRA-2 were unsuitable and not important in both regions. We provide evidence that reanalysis datasets are most suitable for spatiotemporally consistent environmental analysis whereas gauge- or satellite-based products and their combinations are highly variable and may not be applicable in peripheral areas.

## Introduction

Peripheral regions are important refuges for endangered species and pristine vegetation communities. Therefore, numerous conservation areas were established in such locations and long-term monitoring measures have shown to be vital for analysis of global environmental changes^1,2^. Apart from direct anthropogenic impacts, climate variables are the decisive drivers of vegetation variations and strong shifts are predicted in this type of regions^2,3^. Hence, long-term climate datasets are essential for understanding potential ecosystem changes. However, remote regions are often also characterized by poor meteorological infrastructure. This is clearly illustrated by the comparison of national park areas^4^, here defined as IUCN Category II protected areas, and the GPCC full data product^5^, the world’s largest precipitation station data base^6^, that shows that 92% of the area of terrestrial national parks is situated in raster cells without available station data during the last climate normal period 1981–2010 (Appendix 1). There has also been a global decline in the availability of gauge precipitation data since the 1990s^6^. Inadequate or no station data may lead to poor performance of gridded precipitation products and also limits possibilities of independent product evaluation^7–9^. The temporal variations introduce additional analytical uncertainties and although a large number of station-based evaluations exist^10–14^, their results are generally not transferable to regions with poor station data infrastructure^9^. Finally, station-based evaluation studies may suffer from a positive bias by utilizing the same or similar stations as those integrated in the gridded datasets.

Consequently, effective monitoring of the world’s protected areas, and vegetation change in general, requires an assessment of spatial climate products and variables in remote areas and their ability to explain vegetation anomalies. Several studies analyzed the relationship between normalized difference vegetation index (NDVI) variations and selected spatial climate datasets over larger areas^15–19^. However, comprehensive research approaches on the performance of potentially available climate datasets and variables in remote regions are missing. Relevant research on mountain areas is scarce and shows no significant climate-vegetation-relationships or only very low correlations in large parts of these regions even with some of the most popular datasets^16,18,19^. Furthermore, most studies working with medium resolution NDVI data in remote areas concentrate on one single variable or climate dataset and a comparison of the performance of different products is missing^20–24^. Therefore, scientific insights into the reliability of the various spatial climate products for detecting ecological change in regions with insufficient station data are lacking.

In order to close this research gap, and to identify applicable products for analyzing biological changes in peripheral ecosystems with poor meteorological infrastructure, we evaluate a large set of recent state-of-the-art gridded climate products and their ability to explain vegetation anomalies in two remote national parks of Afghanistan. These parks are particularly suitable to assess correlations of vegetation and gridded climate products in remote locations due to their cool, arid to semi-arid setting and the associated coupling of water availability and temperature with vegetation productivity, their complex topography, limited or absent station data, influence of different climate regimes and the very low proportion of direct agricultural activities such as tillage or irrigation^9,25–28^. To cover vegetation changes in complex terrain, we used NDVI anomalies from the moderate resolution MODIS product MOD13Q1^29^ during the period 2001 to 2018. Gridded climate products were selected based on their currentness, their temporal coverage, their station-assessed performance in other regions^9,13,30^, and to encompass various product categories as given in Sun et al.^31^. Additionally, we also include the MODIS snow product MOD10A1^32^ as a spatial climate indicator because snow variables were significantly affecting vegetation anomalies in existing studies^23,33–35^. In summary, we utilize CHIRPS 2.0^36^, CRU TS4.03^37^, ERA5-Land^38^, the GPCC Monitoring Product Version 6^39^, IMERG GPM^40^, MERRA-2^41^ and MOD10A1^32^ to derive test variables for the analysis. Our leading hypothesis is that climate datasets that are less dependent on observational data, such as reanalysis and satellite products, outperform gauge-based raster data in regions with poor station data availability. Furthermore, we expect the high-resolution snow product to be particularly suitable for explaining vegetation anomalies compared to the other, relatively coarse datasets. Therefore, we aim to present a reliable spatial methodology to answer the following main research questions: (i) Which state of the art climate products, climate variables and temporal intervals are suitable to assess vegetation anomalies in remote protected areas?; (ii) How strong is the correlation between climate and vegetation anomalies and what is the areal extent of the correlation?; and (iii) Are the results transferable between regions?

## Methods

### Study area

**Figure 1:**
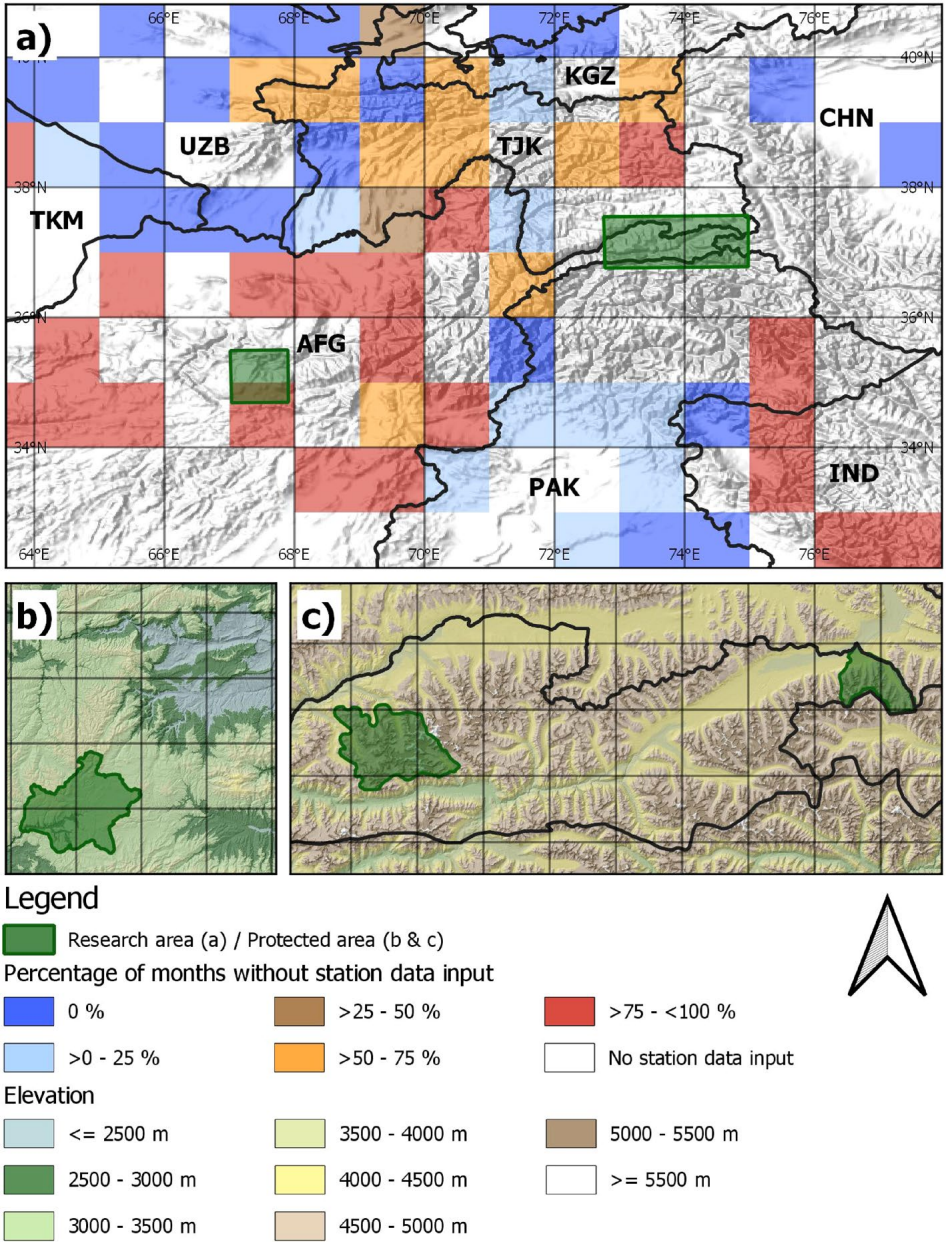
Overview of (**a**) the research areas with an overlay of missing station data in the GPCC Monitoring Product 2001–2018 (EPSG 4326), (**b**) the Band-e-Amir (EPSG 32642 with 20 km grid overlay), and (**c**) the Wakhan research areas (EPSG 32643 with 20 km grid overlay). Monthly missing station data in the lower Band-e-Amir cell is 98% (GPCC data:^39^, relief data:^43,44^). Created using QGIS 3.12 (http://qgis.osgeo.org/)^45^.

We chose protected areas in Wakhan and Band-e-Amir national parks in Afghanistan for this study, as they represent two different environmental settings with diverse influencing climate systems. The Wakhan study area is located in the high mountains of the Pamir and Hindukush with elevations between 2,900 and 6,300 m (Fig. 1). The climate is cold and dry, with an average precipitation of about 200 mm in the valleys, and represents the transition zone from the Westerlies to the Indian summer monsoon with precipitation maxima in spring and summer^9,27^. The region forms the headwaters of a main Central Asian stream, the Amu Darya, rendering it crucial for large scale water supply^42^.

A large number of rare or vulnerable species exists, including the snow leopard (*Panthera uncia*) or the Marco polo sheep (*Ovis ammon polii*), and the diverse flora harbors about 20% of endemic species^28,46^. Varying water availability and temperature regimes lead to well-defined vegetation communities with riparian areas, dwarf-shrub cushion steppes, alpine grasslands and scree vegetation or glaciers at very high altitudes cf.^47^. The selection of the research boundaries, enclosing a total area of 17,000 km^2^, was based on an existing classification raster of vegetation communities which served to exclude non-vegetated areas and detect potential differences between vegetation classes. The Band-e-Amir study area, comprising Afghanistan’s first national park, is located in the west-central Hindukush mountains with elevations from 1,600 m to 4,300 m. In winter and spring, climate is dominated by cyclone offshoots from the west which intensify in spring^48^. During summer months, the entire region is influenced by an almost stationary subtropical high-pressure cell. This causes a characteristic precipitation pattern with a maximum in spring and almost no precipitation during summer months^26^. Vegetation comprises azonal riparian communities and dwarf-shrub cushion steppes reaching up to the highest elevations. Glaciers do not exist in this region. The protected areas provide habitat to several endemic plants, birds and vulnerable mammals, such as the Persian leopard (*Panthera pardus tulliana*), urial (*Ovis orientalis*) or Pallas cat (*Otocolobus manul*), and they include the unique travertine dammed lakes of Band-e-Amir^26,49–51^. Economically, both regions are dominated by pasture farming and only small areas are used for cropping.

### Methodological principle and utilized datasets

Our methodology is based on the well-established concept that vegetation conditions are dependent on the climate^52,53^, in turn leading to close coupling of associated anomalies^18^. This holds especially true for arid to semi-arid regions^54^. In the absence of independent station data, i.e. data that is not included in the gridded products as well, we make use of this knowledge in the evaluation of gridded climate datasets. Where climate datasets accurately represent regional climate conditions, a robust correlation with the vegetation status of large parts of relatively pristine regions can be expected, and such correlation should be observed for different types of datasets^55^. In contrast, climate datasets that do not adequately represent regional climate conditions are unlikely to show significant and robust correlations with temporal variations of vegetation. The level of direct human activities in the selected study areas with the potential to interfere with the relationships of climate and vegetation is low and moreover, would affect all datasets in a similar way. Therefore, the connection of climate variables with vegetation conditions is used as important proxy to assess the suitability of the products in peripheral regions.

The following sections provide an overview of the datasets used in our analyses together with a rationale for their selection. All these datasets were originally obtained with the help of sophisticated creation algorithms. A comprehensive description of the datasets is out of scope of this study and can be found in the cited literature .

#### MODIS NDVI: MOD13Q1

Vegetation data was used as the variable of interest of this study. We selected MODIS NDVI to derive vegetation anomalies as it is an essential and widely used parameter for vegetation monitoring and change detection^56^. To obtain optimal pixel values, we used the MOD13Q1 product. It provides 16-day NDVI values with a resolution of 250 m and a temporal coverage from February 2000 to present and is generated from a daily dataset based on several quality criteria such as low clouds and high NDVI^29^. In analogy to existing anomaly research^57^, to avoid potentially varying effects of different gap-filling approaches due to changing regional performance^58,59^, to minimize possible influence of snow effects and seasonally poor vegetation conditions in mountains^33^, and to maximize the generally low vegetation signal in drylands^60^, we used the mean maximum NDVI during and after the peak of the vegetation period to derive vegetation anomalies. Vegetation peaks occur during June and July in the Band-e-Amir region and during July and August in Wakhan, respectively. As snow effects in summer months are unlikely in the Band-e-Amir region, we also included the month of August in our analysis of this region to achieve consistency with averaging periods of climate variables as described in the corresponding section of this publication. Therefore, the original data was averaged to monthly maximum values, reprojected to the UTM reference system and then clipped to the research areas. Finally, vegetation peak anomalies (Wakhan: July–August, Band-e-Amir: June–August) for each year of the period 2001–2018 were calculated while the mean value of the whole period served as a reference, resulting in a time series of annual vegetation anomalies covering 18 years. Autocorrelation posed a possible issue in this time series—either by inflating correlations due to superposition of global climate patterns or by interfering with significance testing. By applying the method outlined by Hyndman^61^ we were able to confirm for both areas, Wakhan and Band-e-Amir, that autocorrelation did not present itself as a confounding factor.

#### CHIRPS 2.0

The Climate Hazards Group InfraRed Precipitation with Station data (CHIRPS) is a gridded precipitation dataset with a resolution of 0.05° ranging from 1981 to near present that has been developed for data sparse regions by combining a variety of station data, a multitude of satellite observations (thermal infrared, microwave) and forecast models^36^. Existing research shows good performance of the data in other regions^30^. The CHIRPS product was utilized to derive precipitation variables.

#### CRU TS4.03

The Climatic Research Units’ (CRU) most recent product version TS4.03 is a spatial climate dataset with a 0.5° resolution covering the period 1901 to 2018 which is derived from interpolated station values using a long-term climatology^37^. We included this dataset as it is one of the most widely used climate products^19,20,62^. In this study, precipitation and temperature variables were derived from this product.

#### ERA5-Land

At 0.1°, ERA5-Land is a higher resolution version of the European Centre for Medium-Range Weather Forecasts ERA5 climate reanalysis dataset with a coverage from 1981 until present. It has been available since mid-2019 (publication date 2019-Jul-12) and is thus one of the latest reanalysis datasets. Reanalysis data combines models and observational inputs into a consistent product based on physical principles^38^. The starting point of a reanalysis dataset is a numerical weather prediction model. ERA5 uses the Integrated Forecast System cycle 41r2 (Cy41r2), which is combined with a large number of observations (observations of wind, temperature, relative humidity and pressure from in-situ and upper-air soundings, airplane measurements, a multitude of satellites, rain rate from ground-based radar–gauge composite observations since 2009,…) by means of data assimilation techniques^63^. Thereby, a cost function is minimized so that the final analysis is close to the forecast and the observations. For ERA5, a linearized quadratic 4D-Var cost function is used for data assimilation in the atmosphere. For land surface variables, ERA5 implements the land data assimilation system (LDAS), which is connected to 4D-Var^63^. The approach, which is used to construct a long-term climate dataset by combining data of past periods with the current model, is usually referred to as “reanalysis” or “retrospective analysis”^64^. This dataset is a promising candidate for deriving valuable climate parameters of remote mountain regions, because of its high resolution and its foundation in physical laws. The dataset offers a large number of climatic variables. In this analysis, we used temperature, precipitation, snow cover, soil water in the uppermost layer and skin reservoir content. Soil water content is defined as the volume of water from the surface (0 cm) to 7 cm depth in m^3^/m^3^. The skin reservoir includes water on vegetation and on soil, i.e. dew and water intercepted by plants^38^.

#### GPCC Monitoring Product Version 6

The dataset is based on the interpolation of precipitation anomalies from long term climatologies using gauge data in a resolution of 1° and a temporal coverage from 1982 to two months before present^39^. In several evaluation studies, GPCC precipitation products outperformed other datasets^65–67^ and it is considered as the largest precipitation database worldwide^6^. We utilized precipitation variables from the near-realtime monitoring product, referred to as GPCC MP in this manuscript, because the more accurate full data product currently ends in 2016.

#### IMERG GPM

Integrated Multi-satellitE Retrievals for GPM (IMERG) with a resolution of 0.1° and a data range from mid-2000 to present uses multiple passive microwave satellite observations which are adjusted with gauge observations^40^. Different versions of this product exist and we selected the current version 06B final run for our analysis. This product is usually available 3.5 months after the observation and includes gauge analysis, forward and backward morphing. We included precipitation variables from this relatively new satellite product as previous research showed good performance in complex terrain^68^.

#### MERRA-2

The Modern-Era Retrospective analysis for Research and Applications version 2 (MERRA-2) is a reanalysis product with a 0.5° X 0.625° resolution and a data range from 1980 to present^41^. We used the 2d, Monthly mean, Time-Averaged, Single-Level, Assimilation, Surface Flux Diagnostics V5.12.4 product for our analysis (MERRA-2 tavgM_2d_flx_Nx). In analogy to ERA5, it delivers a large range of climate variables. The numerical weather prediction model in this reanalysis dataset is the Goddard Earth Observing System Model, Version 5 (GEOS-5). To combine the model and observations, the Atmospheric Data Assimilation System (ADAS), version 5.12.4 is applied. The analysis is processed with a three-dimensional variational (3DVAR) algorithm based on a Gridpoint Statistical Interpolation (GSI) analysis scheme and a first-guess-at-appropriate-time (FGAT) procedure^69^. The analysis is used to correct the forecast system state with an incremental analysis update (IAU). Compared to previous versions, a major advancement of this product is the additional assimilation of space-based aerosol observations^69^. We used 2 m temperature (referred to as temperature 2 m), surface air temperature (referred to as temperature), precipitation and gauge-corrected precipitation from this product. The uncorrected precipitation is the amount generated by the model without surface observations of precipitation, whereas the corrected precipitation is derived using globally available products based on surface observations^69,70^.

#### MODIS snow: MOD10A1 V6

Snow is considered a major climate factor influencing vegetation growth in cold drylands^23,33,71,72^. The MODIS MOD10A1 V6 product provides daily Normalized Difference Snow Index (NDSI) data from the Terra satellite with a resolution of 500 m from February 2000 until present^32^. To remove cloud cover and other invalid pixels, we performed a simple gap-filling approach. All pixels with > 60% of invalid data during the time series from 2001 to 2018 were excluded from the analysis as they were considered unsuitable for the gap-filling approach. However, only 5% of the area was affected by this criterion in the Wakhan region and no pixels with > 60% missing data were found in the Band-e-Amir region. In the remaining pixels, gaps were filled by a linear interpolation method. Invalid cells at the beginning and end of the time series were filled using the closest available value. Although several gap-filling approaches exist, we selected this approach due to its simplicity, transferability, user-friendliness, independence and effectiveness cf.^73^. After gap-filling, we derived two additional snow metrics in addition to the raw NDSI, fractional snow cover (FSC) and snow cover duration (SCD). FSC was calculated based on the formula given in Salomonson and Appel^74^ as:

1 $$ {\text{FSC}} = - 0.01 + 1.45 *{\text{NDSI}} $$

Values below zero and above 100 were truncated to 0% and 100%. SCD was calculated as the sum of days with NDSI above 0.2 following Riggs et al.^75^. All daily estimates were finally aggregated to monthly values. In the near future, a gap-filled version of the product, MOD10A1F, will be available from the NASA Distributed Active Archive Center (DAAC) at the National Snow and Ice Data Center^73^.

All climate datasets were reprojected and resampled to match the NDVI dataset using nearest neighbor interpolation. While different resampling techniques exist, we selected this approach to preserve the original dataset values^76^, and avoid variations related to resampling as stated in existing research^77^.

#### Vegetation classification

Vegetation classifications are frequently used to support studies on vegetation-climate relationships^19,22,62^. As substantial areas in the Wakhan region are permanently covered by glaciers, snow or rocks, information on vegetated areas from an existing supervised classification was used to exclude such unvegetated regions. The respective classification utilized 370 field mapped ground truthing points and cloud free, Sentinel 2 satellite images from 2018 with a random forest classification approach and showed a validated overall accuracy of 92%^78,79^. The original classification was reprojected and resampled to match the NDVI dataset. Finally, all non-vegetated pixels of the spatial datasets were excluded from the analysis in the Wakhan region. Furthermore, the classification was used to test for potential differences between riparian vegetation, dwarf-shrub cushion steppes and alpine grasslands. In the Band-e-Amir region, no classification was used as no glaciers or permanent snow exist and the whole area is potentially vegetated.

### Selection of averaging periods for climate variables

Vegetation response to climate variations is characterized by significant temporal and spatial variability^19^. Therefore, ideal temporal averaging periods depend on the specific environment and the derivation must be based on individual vegetation-climate interrelationships of the research area. A frequently used variable is the hydrological year^21,23,35^. We slightly adapted this to match vegetation growth and defined the hydrological year in our research area as the 12-month period before the vegetation peak. Therefore, hydrological year anomalies were calculated based on averages from July of the previous year until June of the year in question, i.e. the year of the vegetation anomaly. By controlling soil moisture availability until summer and determining the start of the growing season, winter and spring climate conditions may be among the most important factors influencing vegetation growth^16,19,34^. We calculated several anomaly periods that consider the respective seasons accordingly: winter half year (November to April), Spring (March–May) and the transition period February to March. Finally, summer was also reported as relevant for Central Asian ecosystems^16,72^. Hence, we included the summer period (June–August) and the combined spring–summer period (March-August). MODIS snow variable anomalies were not calculated for time periods including summer months. In addition to influences of the current year, long-term responses of vegetation to climate variations exist, especially in semi-arid regions^16,80,81^. Therefore, we also included two-year averages of all seasonal climate variables. All anomalies were calculated with the respective average of all years as a reference value.

### Statistical analysis

The multitude of analyzed climate datasets, variables and the different temporal periods led to a total number of 156 potential features for analyzing vegetation anomalies. Most existing studies are either based on pixel correlations^16,19,23,33^ or correlation analysis of regionally averaged anomalies^62^. As both approaches provide valuable information, the former on the spatial performance and pattern, the latter on the strength of the overall correlation, we combined respective approaches. To assess which products are suitable for explaining vegetation anomalies for each region in general, we performed correlation analyses using area averaged anomalies. However, hypothesis testing with the large number of potential features leads to a multiple testing problem as 200 independent hypothesis tests at the 5% significance level would lead to the expectation of 10 false rejections of the null hypothesis. Therefore, we applied the Benjamini–Hochberg procedure^82^ to compute adjusted *p* value thresholds for all climate variables controlling the false-discovery rate (FDR) at a level of 5%. The respective approach was successfully applied by existing remote sensing studies^83,84^. To asses uncertainty of the correlation coefficients, we computed bootstrap bias-corrected and accelerated (BCa) 95% confidence intervals^85^ using 2500 replicates as respective method showed good performance in comparable studies^86,87^. A per pixel analysis was conducted in addition to area averaged significance tests to derive information on the spatial performance of variables following the methodology of Abdi et al.^88^. Thereby, we computed correlation and *p* values for each cell and summarized the percentage of significant pixels (*p* ≤ 0.05) in relation to the analyzed total area. By combining both approaches, we aim to achieve a consistent picture on the temporal and spatial performance of climate variables and their ability to explain vegetation differences.

Climate product variables are considered as good explanatory variables for vegetation anomalies if they show a significant overall correlation controlled at a FDR < 5%, and if the correlation is significant in the majority (≥ 50%) of analyzed pixels. Respective variables are referred to as *highly suitable variables* in this manuscript. In the Wakhan research area, we conducted this analysis separately for the vegetation classes riparian vegetation, dwarf-shrub cushion steppes and alpine grasslands to assess community-based differences.

We selected Pearson’s correlation coefficient as the main statistical method as preliminary studies in comparable environments suggested a linear relationship between vegetation anomalies and climate data^16,19,23,34,53,81^. However, some studies state a non-linear response of vegetation to climate variations^18^. To also consider non-linear interlinkages, we repeated the analysis using Spearman rank correlation to assess potential differences of the different methods. As results are expected to be variable due to the high dimensionality and the comparably low number of available years, we additionally assessed the stability of suitable datasets using different variable selection approaches in a prediction context. We applied two different methods in this study, as a comparison of different approaches is suggested if modeling is performed with high dimensional data^89^. One approach, which is methodologically comparable to our correlation analysis, is the calculation of models with single stepwise forward selection based on Pearson’s correlation and 100 repeated, threefold cross-validation cf.^84^. Thereby, we quantified the proportion of the selection as the best variable in relation to the total number of selections. Additionally, we chose the Boruta algorithm to assess variable importance and selection, as it is considered as the most powerful approach in high dimensional settings by recent studies^90^. In this approach, variables are randomly shuffled to create *shadow variables*, and importance scores are then calculated using these *shadow variables* and original predictors, i.e. the climate variables, to predict vegetation anomalies with a random forest regression^91^. Finally, only variables that have significantly higher scores than the *shadow variables* are considered as important^92^. We used the R package Boruta with a maximal number of importance source runs of 500 and a *p* value of 0.05. As the process is variable due to stochasticity of the random forest classifier^91^, we repeated the whole procedure 500 times and quantified the percentage of confirmed selections and the mean importance of selected variables over all repetitions.

## Results

### Overall correlations and highly suitable variables

The comparison of correlation methods showed that *highly suitable variables* for analyzing vegetation anomalies were almost identical in the Spearman and Pearson approach (Tables 1, 2, Appendix 2, Appendix 3). Generally, more variables were considered as highly suitable with the latter, linear method. Therefore, we focus on the presentation of results using the Pearson correlation in this section. Results showed that only variables of four products, MERRA-2, ERA5-Land, MODIS MOD10A1 and CRU, may be considered as *highly suitable* for vegetation analysis in the Wakhan research area (Table 1).

**Table 1:** Highly suitable variables (significant values controlled at a FDR < 5% and ≥ 50% of analyzed pixels show significant correlation) and evaluation metrics in the Wakhan study region. Descending order based on percentage of significant pixels.

Dataset	*p*	R (confidence interval)	% significant pixels
MERRA-2 precipitation/ Hydrological-year	0.0000	0.82 (0.6/0.91)	78
MERRA-2 precipitation/Winter half-year (Nov-Apr)	0.0001	0.79 (0.4/0.91)	74
MERRA-2 precipitation/Spring–Summer (Mar-Aug)	0.0008	0.72 (0.49/0.85)	71
MERRA-2 precipitation/Spring (Mar-May)	0.0014	0.69 (0.45/0.82)	68
ERA5-Land precipitation/Spring–Summer (Mar-Aug)	0.0012	0.7 (0.39/0.84)	66
MODIS-NDSI/Spring (Mar-May)	0.0013	0.7 (0.3/0.88)	65
MODIS-FSC/Spring (Mar-May)	0.0015	0.69 (0.31/0.89)	65
MODIS-SCD/Spring (Mar-May)	0.0012	0.7 (0.32/0.9)	64
ERA5-Land soil water/Summer (Jun-Aug)	0.0010	0.71 (0.46/0.84)	60
MODIS-NDSI/Transition (Feb-Mar)	0.0021	0.67 (0.26/0.87)	60
MODIS-FSC/Transition (Feb-Mar)	0.0025	0.67 (0.25/0.87)	57
MERRA-2 precipitation/Transition (Feb-Mar)	0.0075	0.61 (0.16/0.82)	56
CRU precipitation/Winter half-year (Nov-Apr)	0.0025	0.67 (0.29/0.87)	54
ERA5-Land skin-reservoir content/Spring–Summer (Mar-Aug)	0.0014	0.69 (0.29/0.86)	53
MODIS-FSC/Spring (Mar-May) two-year average	0.0078	0.62 (0.15/0.85)	52
ERA5-Land precipitation/Spring (Mar-May)	0.0082	0.6 (0.23/0.81)	52
MODIS-NDSI/Spring (Mar-May) two-year average	0.0083	0.62 (0.14/0.85)	52
MODIS-SCD/Spring (Mar-May) two-year average	0.0072	0.63 (0.15/0.85)	52

**Table 2:** Highly suitable variables (significant values controlled at a FDR < 5% and ≥ 50% of analyzed pixels show significant correlation) and evaluation metrics in the Band-e-Amir study region. Descending order based on percentage of significant pixels.

Dataset	*p*	R (confidence interval)	% significant pixels
ERA5-Land precipitation/ Hydrological-year	0.0000	0.92 (0.72/0.97)	97
MERRA-2 precipitation/ Hydrological-year	0.0000	0.85 (0.66/0.94)	96
ERA5-Land soil water/Summer (Jun-Aug)	0.0000	0.88 (0.7/0.94)	95
ERA5-Land soil water/Spring–Summer (Mar-Aug)	0.0000	0.87 (0.64/0.94)	95
MERRA-2 precipitation/Spring–Summer (Mar-Aug)	0.0001	0.81 (0.46/0.93)	95
ERA5-Land precipitation/Spring–Summer (Mar-Aug)	0.0001	0.8 (0.59/0.91)	95
MERRA-2 precipitation/Winter half-year (Nov-Apr)	0.0000	0.82 (0.54/0.94)	94
CRU precipitation/ Hydrological-year	0.0002	0.77 (0.44/0.9)	93
ERA5-Land precipitation/Winter half-year (Nov-Apr)	0.0002	0.76 (0.37/0.92)	93
MERRA-2 precipitation/Spring (Mar-May)	0.0001	0.78 (0.41/0.92)	92
ERA5-Land temperature/Spring–Summer (Mar-Aug)	0.0002	− 0.76 (− 0.9/ − 0.43)	90
MERRA-2 temperature/Spring–Summer (Mar-Aug)	0.0004	− 0.75 (− 0.89/ − 0.47)	89
MERRA-2 precipitation/Transition (Feb-Mar)	0.0006	0.73 (0.27/0.88)	88
ERA5-Land skin-reservoir content/Spring–Summer (Mar-Aug)	0.0000	0.87 (0.6/0.95)	85
MERRA-2 temperature 2 m/Spring–Summer (Mar-Aug)	0.0010	− 0.71 (− 0.87/ − 0.43)	85
CRU precipitation/Winter half-year (Nov-Apr)	0.0025	0.67 (0.29/0.87)	84
IMERG precipitation/ Hydrological-year	0.0002	0.76 (0.46/0.89)	83
ERA5-Land skin-reservoir content/Spring (Mar-May)	0.0000	0.85 (0.58/0.94)	80
ERA5-Land temperature/ Hydrological-year	0.0019	− 0.68 (− 0.86/ − 0.35)	80
ERA5-Land soil water/Spring (Mar-May)	0.0001	0.78 (0.48/0.91)	80
ERA5-Land skin-reservoir content/Summer (Jun-Aug)	0.0019	0.68 (0.29/0.84)	79
MERRA-2 temperature/Spring (Mar-May)	0.0030	− 0.66 (− 0.85/ − 0.3)	79
ERA5-Land temperature/Spring (Mar-May)	0.0041	− 0.64 (− 0.85/ − 0.18)	77
IMERG precipitation/Winter half-year (Nov-Apr)	0.0011	0.7 (0.32/0.89)	77
ERA5-Land snow/ Hydrological-year	0.0005	0.73 (0.38/0.85)	76
ERA5-Land soil water/ Hydrological-year	0.0008	0.72 (0.38/0.85)	76
ERA5-Land precipitation/Spring (Mar-May)	0.0030	0.66 (0.29/0.85)	75
CRU precipitation/Spring–Summer (Mar-Aug)	0.0022	0.67 (0.35/0.83)	74
MERRA-2 temperature 2 m/Spring (Mar-May)	0.0046	− 0.64 (− 0.83/ − 0.29)	74
ERA5-Land temperature/Summer (Jun-Aug)	0.0055	− 0.63 (− 0.81/ − 0.03)	72
ERA5-Land skin-reservoir content/ Hydrological-year	0.0000	0.82 (0.56/0.92)	71
CRU precipitation/Spring (Mar-May)	0.0030	0.66 (0.32/0.82)	71
ERA5-Land precipitation/Summer (Jun-Aug)	0.0046	0.64 (0.26/0.82)	70
ERA5-Land snow/Spring–Summer (Mar-Aug)	0.0043	0.64 (0.2/0.88)	65
ERA5-Land snow/Spring (Mar-May)	0.0046	0.64 (0.2/0.88)	65
ERA5-Land snow/Winter half-year (Nov-Apr)	0.0016	0.69 (0.32/0.82)	65
IMERG precipitation/Transition (Feb-Mar)	0.0042	0.64 (0.29/0.83)	65
ERA5-Land temperature/Spring–Summer (Mar-Aug) two-year average	0.0098	− 0.61 (− 0.82/ − 0.16)	62
MERRA-2 temperature/ Hydrological-year	0.0143	− 0.57 (− 0.8/ − 0.08)	61
CRU temperature/Spring (Mar-May)	0.0124	− 0.58 (− 0.83/ − 0.11)	60
MODIS-NDSI/Spring (Mar-May)	0.0049	0.63 (0.28/0.81)	58
MODIS-NDSI/ Hydrological-year	0.0067	0.63 (0.16/0.85)	58
MODIS-FSC/Spring (Mar-May)	0.0055	0.63 (0.26/0.81)	57
MODIS-FSC/ Hydrological-year	0.0066	0.63 (0.16/0.85)	56
MODIS-SCD/Spring (Mar-May)	0.0059	0.62 (0.23/0.82)	55
MERRA-2 temperature/Spring–Summer (Mar-Aug) two-year average	0.0146	− 0.58 (− 0.79/ − 0.17)	55
MODIS-NDSI/Transition (Feb-Mar)	0.0094	0.59 (0.25/0.78)	52
MODIS-NDSI/Winter half-year (Nov-Apr)	0.0110	0.6 (0.13/0.83)	51
MODIS-FSC/Transition (Feb-Mar)	0.0107	0.59 (0.23/0.78)	51

In total, 18 variables were considered as good explanatory variables, including eight from the MOD10A1 product, five from MERRA-2, four from ERA5-Land and one from CRU. All variables showed positive correlations and were directly or indirectly associated with precipitation. Temperature anomalies did not show significant correlations. Reanalysis datasets were ranked highest in both correlation coefficient and percentage of significant pixels. MERRA-2 precipitation during the hydrological year performed best with a R of 0.82 with 95% BCa confidence interval [0.6/0.91] and with 78% of the analyzed pixels showing a significant correlation. Other MERRA-2 precipitation variables showed similar correlations. ERA5-Land and MODIS snow variables also indicated good performance for explaining vegetation anomalies. CRU precipitation was also a *highly suitable variable* in the research area. CHIRPS, GPCC MP and IMERG, as well as the gauge corrected variables of MERRA-2 were not significant and did not provide *highly suitable variables* in the Wakhan research area. Temporal periods comprised different time sections whereby most included spring months.

The Band-e-Amir analysis resulted in 49 *highly suitable variables* (Table 2). Thereby, five were derived from the CRU product, 22 from ERA5-Land, three from IMERG, 11 from MERRA-2 and eight from the MODIS snow product. The majority of suitable variables (n = 37) were associated with precipitation and positively correlated with vegetation anomalies. However, 12 temperature variables indicated negative correlations with vegetation. Reanalysis datasets resulted in the highest correlation coefficients and percentage of significant pixels. ERA5-Land precipitation during the hydrological year performed best with a R of 0.92 with 95% BCa confidence interval [0.72/0.97] and 97% of the pixels showing a significant correlation. MERRA-2 precipitation products resulted in slightly weaker performance. CRU and IMERG showed lower correlation values but most pixels were significant. MODIS snow variables were found to be highly suitable but regions with significant correlations were smaller. Regarding temperature variables, ERA5-Land and MERRA-2 resulted in the highest correlations and the largest percentage of significant raster cells. In terms of the temporal periods, spring months were again included in the majority of the *highly suitable variables*. CHIRPS, GPCC MP and the gauge corrected MERRA-2 precipitation variables were not among the important explanatory variables.

Regarding the variation in a prediction context, the occurrences of datasets and variables averaged over all repeated stepwise variable selections and folds (n = 300) showed a considerable majority of the best datasets in both regions (Table 3). In Wakhan, 61% of the best variables were MERRA-2 precipitation parameters and in Band-e-Amir, 78% were ERA5-Land hydrological parameters. The other selected product variables were similar to the overall correlation analysis, but the percentage of selection was low.

**Table 3:** Percentage of the 10 most frequently selected climate variables averaged over all repetitions and folds (n = 300) according to the single stepwise forward selection.

Wakhan datasets	Selection %	Band-e-Amir Datasets	Selection %
MERRA-2 precipitation/ Hydrological-year	36	ERA5-Land precipitation/ Hydrological-year	57
MERRA-2 precipitation/Winter half-year (Nov-Apr)	20	ERA5-Land soil water/Summer (Jun-Aug)	10
MERRA-2 precipitation/Spring–Summer (Mar-Aug)	5	MERRA-2 precipitation/ Hydrological-year	8
ERA5-Land precipitation/Spring–Summer (Mar-Aug)	4	MERRA-2 precipitation/Spring–Summer (Mar-Aug)	5
ERA5-Land snow/ Hydrological-year	4	ERA5-Land soil water/Spring–Summer (Mar-Aug)	5
CRU precipitation/Spring (Mar-May)	4	ERA5-Land skin-reservoir content/Spring–Summer (Mar-Aug)	5
CRU precipitation/Spring–Summer (Mar-Aug)	3	MERRA-2 precipitation/Winter half-year (Nov-Apr)	4
ERA5-Land soil water/Summer (Jun-Aug)	3	MERRA-2 precipitation/Spring (Mar-May)	2
CRU precipitation/Transition (Feb-Mar)	2	ERA5-Land precipitation/Winter half-year (Nov-Apr)	1
MODIS-SCD/Spring (Mar-May) two-year average	2	ERA5-Land skin-reservoir content/Spring (Mar-May)	1

The 500-repeated Boruta algorithm showed that reanalysis datasets had the highest importance and were considered as important in all repetitions in both research areas (Table 4). In Band-e-Amir, the IMERG dataset was also among the most important products and temperature variables of reanalysis datasets were listed as well. Furthermore, CRU precipitation was considered as important variable in both regions. In Wakhan, CRU temperature and, to a lesser extent, MODIS snow anomalies were also among the important variables. GPCC, CHIRPS and the bias corrected version of MERRA-2 were not listed as important variables.

**Table 4:** Mean importance and percentage of confirmed selections averaged over 500 repetitions of the Boruta algorithm with vegetation anomalies as dependent variable.

Wakhan Datasets	Mean importance	Selection %	Band-e-Amir Datasets	Mean importance	Selection %
MERRA-2 precipitation/Spring–Summer (Mar-Aug)	6.61	100	ERA5-Land precipitation/ Hydrological-year	5.68	100
MERRA-2 precipitation/ Hydrological-year	5.69	100	IMERG precipitation/ Hydrological-year	5.55	100
ERA5-Land soil water/Summer (Jun-Aug)	5.15	100	MERRA-2 precipitation/Spring–Summer (Mar-Aug)	5.51	100
CRU precipitation/Spring–Summer (Mar-Aug)	4.5	100	MERRA-2 precipitation/ Hydrological-year	5.04	100
ERA5-Land soil water/Spring–Summer (Mar-Aug)	4.5	100	MERRA-2 precipitation/Spring (Mar-May)	4.88	100
CRU precipitation/Spring (Mar-May)	4.28	100	ERA5-Land soil water/Summer (Jun-Aug)	4.48	100
MERRA-2 precipitation/Spring (Mar-May)	4.04	100	MERRA-2 precipitation/Winter half-year (Nov-Apr)	4.11	100
CRU temperature/Spring (Mar-May) two-year average	3.76	100	ERA5-Land precipitation/Spring–Summer (Mar-Aug)	3.89	100
MERRA-2 precipitation/Winter half-year (Nov-Apr)	3.63	98.8	MERRA-2 precipitation/Transition (Feb-Mar)	3.71	100
MERRA-2 precipitation/Transition (Feb-Mar) two-year average	2.72	85.8	IMERG precipitation/Spring–Summer (Mar-Aug)	3.53	100
ERA5-Land precipitation/Spring–Summer (Mar-Aug)	2.41	86	ERA5-Land skin-reservoir content/Spring–Summer (Mar-Aug)	3.18	100
CRU temperature/Spring–Summer (Mar-Aug) two-year average	2.29	83.4	ERA5-Land soil water/Spring–Summer (Mar-Aug)	3.16	100
MERRA-2 precipitation/Spring–Summer (Mar-Aug) two-year average	2.22	80.8	IMERG precipitation/Winter half-year (Nov-Apr)	2.91	100
ERA5-Land snow/Spring (Mar-May)	2.19	74.2	MERRA-2 temperature/Spring–Summer (Mar-Aug)	2.74	99
ERA5-Land skin-reservoir content/Spring–Summer (Mar-Aug)	2.13	76	ERA5-Land soil water/ Hydrological-year	2.73	100
ERA5-Land skin-reservoir content/Spring (Mar-May)	1.52	26.4	CRU precipitation/ Hydrological-year	2.71	99.6
ERA5-Land snow/ Hydrological-year	1.4	2	ERA5-Land soil water/Spring (Mar-May)	2.7	100
MODIS-SCD/Spring (Mar-May)	1.08	13.4	ERA5-Land skin-reservoir content/ Hydrological-year	2.6	100
ERA5-Land precipitation/Spring–Summer (Mar-Aug) two-year average	0.99	0.6	CRU precipitation/Spring–Summer (Mar-Aug)	2.46	99
ERA5-Land skin-reservoir content/Summer (Jun-Aug)	0.87	2.2	CRU precipitation/Spring (Mar-May)	2.38	98
MODIS-NDSI/Spring (Mar-May)	0.76	2.6	ERA5-Land skin-reservoir content/Spring (Mar-May)	2.27	92
MODIS-FSC/Spring (Mar-May)	0.7	1.4	ERA5-Land temperature/Spring–Summer (Mar-Aug)	2.2	86.2
ERA5-Land snow/Spring–Summer (Mar-Aug)	0.42	0.2	MERRA-2 precipitation/Summer (Jun-Aug)	2.11	16.2

**Figure 2:**
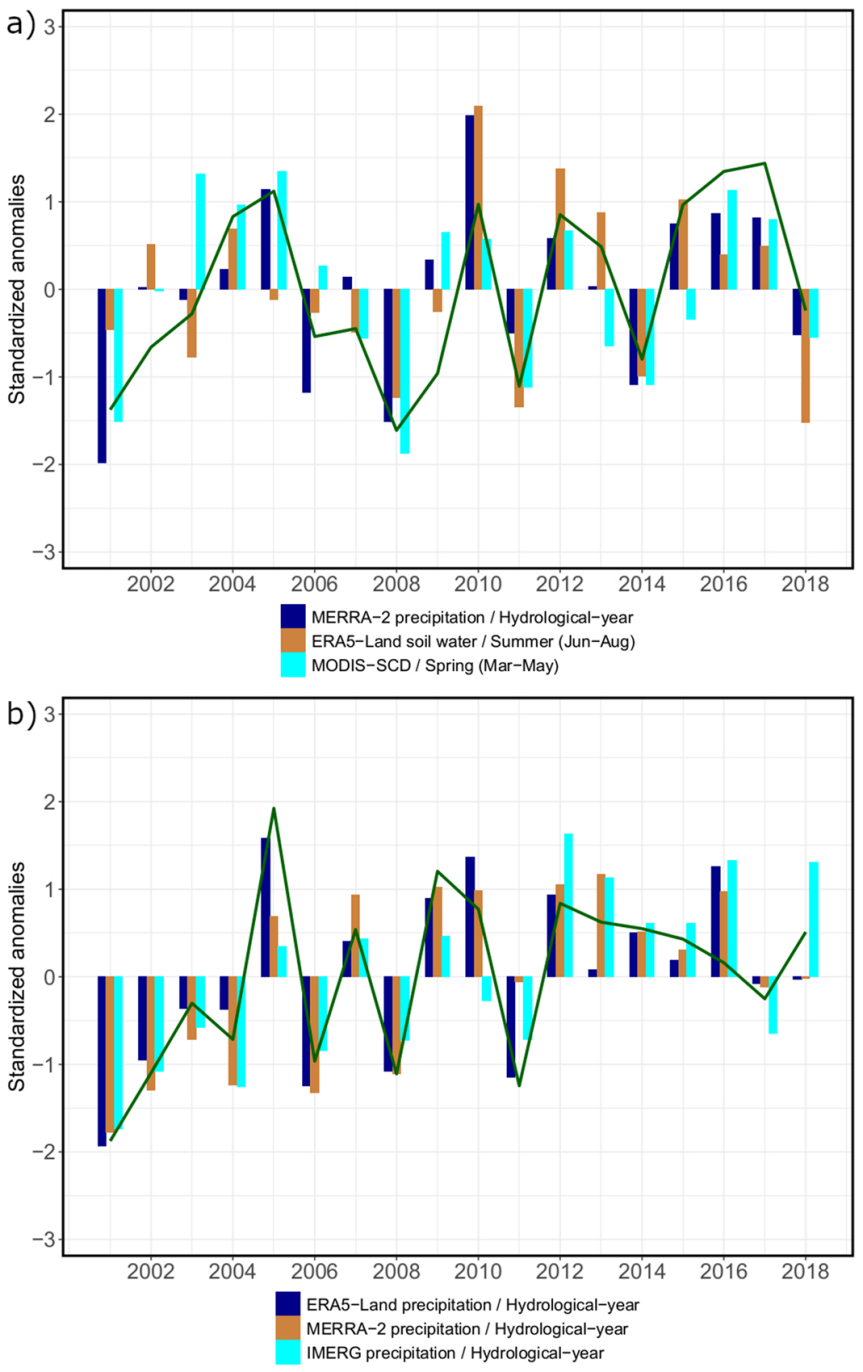
Standardized anomalies of the three highest correlated variables selected from different climate data products (colored bars) compared to MODIS NDVI anomalies (green line) in the Wakhan region (**a**) and the Band-e-Amir region (**b**). Created using R 4.0.3. (https://www.R-project.org/)^93^.

The visual comparison of anomalies of different climate data products with the highest correlations showed relatively similar directions to NDVI anomalies in most years although magnitudes were different between the products (Fig. 2). In general, consistency between climate data and NDVI was considerably higher in the Band-e-Amir region. Regarding climate datasets in Wakhan, there was higher agreement between the reanalysis datasets compared to MODIS snow cover. In Band-e-Amir, anomaly directions of datasets were almost identical with two exceptions of the IMERG product.

### Spatial correlation patterns

**Figure 3:**
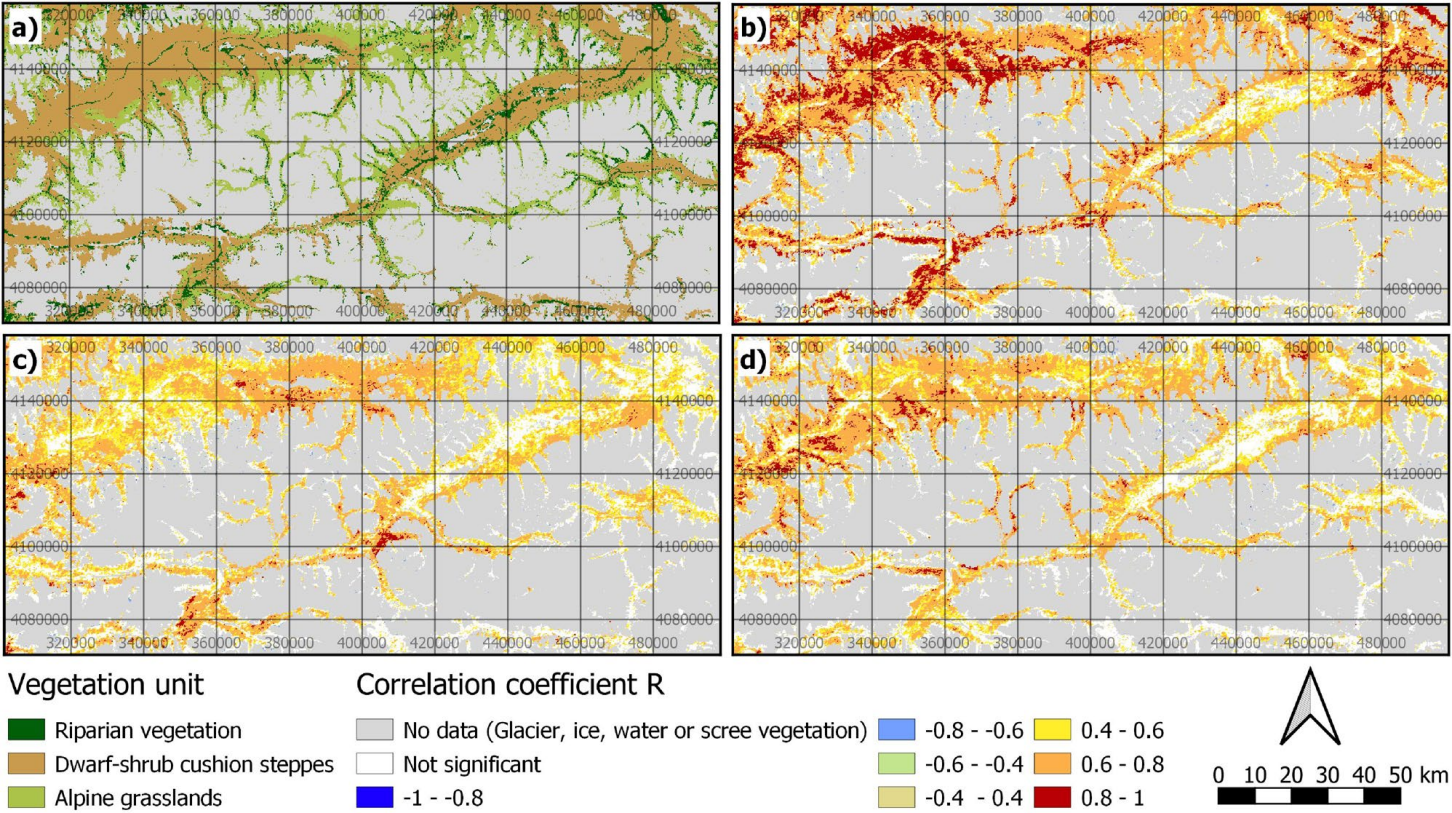
Vegetation units (**a**), significant Pearson’s correlation of MERRA-2 precipitation/Hydrological-year (**b**), ERA5-Land precipitation/Spring–Summer (Mar-Aug) (**c**), and MODIS-NDSI/Spring (Mar-May) (**d**) in the Wakhan region (projection: UTM zone 43 N). Created using QGIS 3.12 (http://qgis.osgeo.org/)^45^.

**Figure 4:**
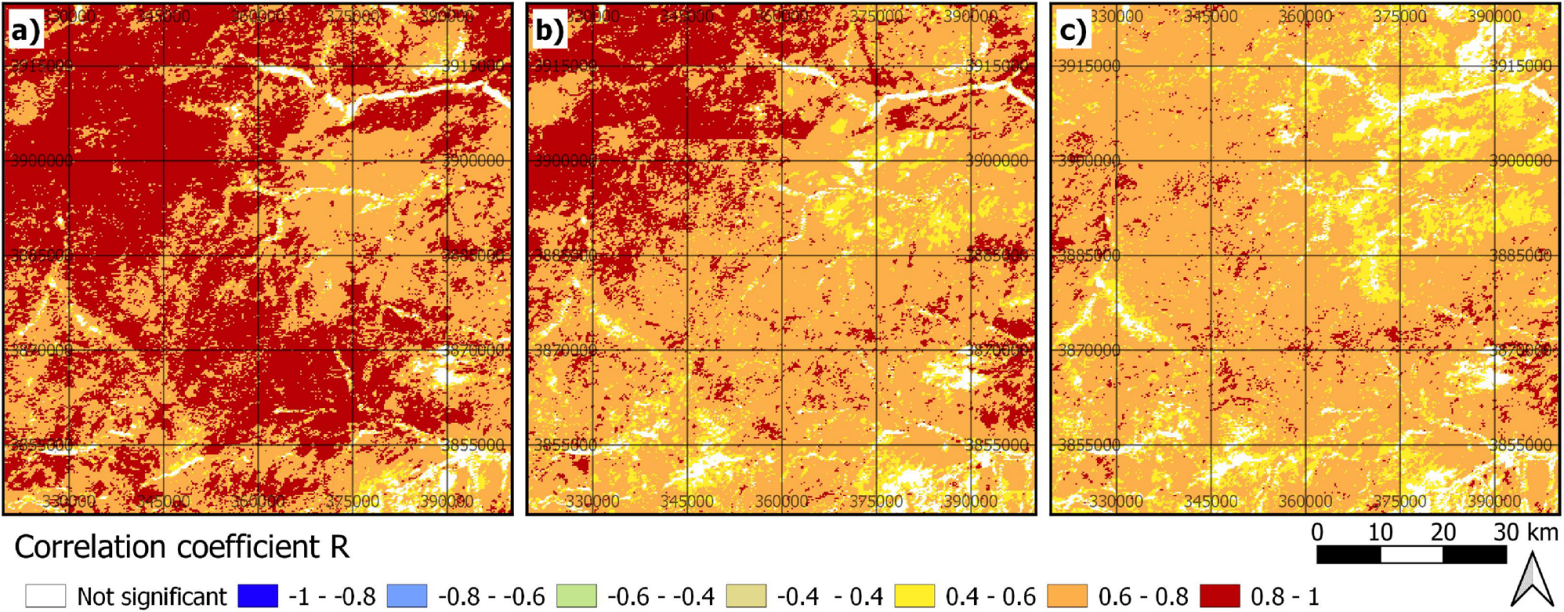
Significant Pearson’s correlation of ERA5-Land precipitation/ Hydrological-year (**a**), MERRA-2 precipitation/ Hydrological-year (**b**), and CRU precipitation/ Hydrological-year (**c**) in the Band-e-Amir region (projection: UTM zone 42 N). Created using QGIS 3.12 (http://qgis.osgeo.org/)^45^.

The spatial pattern of significant values generally showed large agreement regarding the non-significant areas between the three best variables selected from different products in both research areas (Figs. 3, 4). Most non-significant regions were located in valleys and near rivers. In the Wakhan region, higher correlations were found for the northwestern area with lower values in the southeast. Furthermore, the MODIS snow product indicated more non-significant areas in the eastern valleys compared to the other products. In the Band-e-Amir region, reanalysis datasets showed much higher correlations in many areas. Generally, lower correlations were found in lowlands and basins in the northeast, southeast and southwest. Higher correlations were generally found in the northwest and central regions.

The separate analysis of different vegetation units in the Wakhan region illustrated some community related differences with 32 *highly suitable variables* in alpine grasslands, 15 in dwarf-shrub cushion steppes and only four in riparian communities (Appendix 4). Many variables were similar to the combined analysis but in alpine grasslands, CRU temperature was also among the *highly suitable variables* with a significant negative correlation (e.g. Pearson’s r of − 0.66 for CRU temperature/Spring two-year average). Furthermore, significant correlations with NDVI anomalies were found on larger areas in this vegetation unit, especially with snow variables (e.g. 75% of significant pixels with MODIS-NDSI/Spring). In dwarf-shrub cushion steppes, variables were similar to the overall analysis with a slightly better performance of the best variables (MERRA-2 precipitation hydrological-year r = 0.84 and 79% significant pixels). Main differences were found for riparian communities where only MERRA-2 precipitation variables were considered *highly suitable*.

## Discussion

This is the first research approach that systematically assesses and compares the potential of multiple state-of-the-art gridded climate datasets for analyzing vegetation change in peripheral protected areas with lacking meteorological infrastructure. Results clearly show that reanalysis datasets are most suitable in respective regions and that this outcome is consistent between different research areas and between different cross validation folds. These findings are supported by some studies successfully utilizing reanalysis datasets in ecological^21,94–96^, hydrological^97^ or epidemiological research^98^. Furthermore, Dee et al.^99^ consider reanalysis products as the most accurate and homogenous datasets in recent decades which is in agreement with this study. Reanalysis datasets may also provide advantages in regions with a considerable proportion of snow among total precipitation as they are not affected by undercatch errors that frequently lead to measurement errors between 20 to 50% in station data^9,100^. However, comparisons between different products are scarce and most studies used station-based datasets such as CRU or GPCC to analyze vegetation anomalies^16,19,20,62,80,101,102^. This widespread utilization of station-based datasets for ecological research may be due to the existence of numerous research approaches that evaluated raster products with gauge data and stated good associated performances^11,13,65–67,103^. However, these studies may be positively biased due to overlap of validation and incorporated stations and by ignoring temporal variations in gridded datasets^9^. Although station-based interpolations may constitute important predictors for vegetation change in many regions, which was also partly valid in this study for CRU TS4.03, the presented analysis indicates that those datasets are less suitable for vegetation anomaly analysis in peripheral regions compared to reanalysis datasets. In both research areas, variables of the GPCC MP are unsuitable for analyzing vegetation anomalies. However, if it is required to analyze long time spans of 100 years or more, only gauge datasets are available. The presented findings provide evidence that CRU TS4.03 is better suited for vegetation analysis in data-poor regions of Central Asia compared to the GPCC MP but this may be regionally variable. Our results also indicate that the MODIS snow product provides important climate variables in cold drylands which is supported by research for other regions^23,33^ whereby significant correlations are higher and more extensive in the Wakhan region of this study. Although respective variables were also highly suitable for analyzing vegetation anomalies in the Band-e-Amir region, the considerably lower correlation values imply strong regional differences. However, the fine spatial resolution of the MODIS snow product in comparison to the other, relatively coarse datasets suggests large potential of this variable for finer scale analyses. The derivation of snow cover using time series of moderate to high resolution sensors with appropriate temporal resolution, such as Sentinel-1 or Sentinel-2^104,105^, may be promising for deriving climate indicators in the future.

Variables of the satellite based IMERG dataset revealed only weak, non-significant relationships with vegetation anomalies and thus are unsuitable for explaining vegetation variations in the Wakhan region. However, in the Band-e-Amir area, this product was not among the highest correlated variables, but it is a suitable climate dataset. The latter result is backed by other studies that successfully applied IMERG GPM data for drought monitoring or productivity analysis^106,107^. The unsuitability of IMERG GPM in the high mountain Wakhan region may be explained by the worsened performance and precipitation underestimation in higher altitudes as outlined by Lu et al.^108^. Surprisingly, the CHIRPS 2.0 data; which was explicitly designed for monitoring global environmental change in data sparse regions and which showed good results in Africa^30,109^, is not suitable for analyzing vegetation changes in both of our research areas. A major reason for this may be the limited ability of the algorithm to detect snowfall as shown by Bai et al. in China^110^. Furthermore, this product combines two data types that were not or only partly suitable in the respective regions, as it uses interpolated station data similar to GPCC, and the satellite based Tropical Rainfall Measuring Mission Multi-satellite Precipitation Analysis which is the predecessor to IMERG GPM^109^. Therefore, our analysis indicates that the performance of CHIRPS may be problematic in some remote mountain areas outside of Africa. Another remarkable result is that the bias-corrected version of MERRA-2 is unsuitable for vegetation change analysis in our study as opposed to the uncorrected version that ranked among the best datasets. This is in contrast to existing research which showed improvement of reanalysis data for hydrological analysis after observation based bias correction in North America^111^. However, the density of observation data is comparably high in North America and hence, bias correction approaches are more likely to improve reanalysis data in those areas. In regions with poor meteorological infrastructure, where only a small number of stations is available that are potentially located far from the raster pixel in question, the correction algorithm may potentially distort the physically based reanalysis model. Furthermore, mountain regions may be particularly susceptible to errors based on small station numbers as many regions are not sufficiently represented in complex topography^9^.

Regarding the different variables, precipitation was positively correlated to vegetation anomalies and can be considered as the most suitable variable for predicting vegetation anomalies. This result is typical for drylands where vegetation growth is usually constrained by precipitation whereas it is more resilient to temperature anomalies^18,19^. However, temperature anomalies are important in some regions and negative correlations indicate indirect effects on plant available water by increasing snowmelt and evapotranspiration. These findings partly contradict results of other regions where spring temperature showed a positive correlation with vegetation but was in agreement concerning summer temperatures with negative correlations^62^. This shows that temperature effects on vegetation may be highly variable on spatial and temporal scales^34^. The biological importance of the spring season is supported by other results and underlines that different time periods influence vegetation variations^19,34^. Similarly, results also showed that long term anomalies are important in explaining yearly vegetation anomalies in dryland regions^16^. The yearly precipitation sum before the vegetation peak, i.e. the hydrological year, was the most important temporal period which shows that all seasons have impacts on vegetation anomalies. Their combination in anomaly calculation may therefore contribute to vegetation change analysis despite missing correlation of some individual seasons with vegetation.

The 100 repeated threefold cross validation and the 500-repeated Boruta algorithm showed that there was some variability of the best variables when different years are considered, but in most folds and repetitions, the same products were selected as in the overall correlation and spatial performance analysis. This indicates that the results are robust over various yearly feature sets. However, it is important to consider notable uncertainties of the correlation coefficients due to the limited number of years, as indicated by the 95% BCa confidence intervals with wide intervals for variables with lower overall correlation. Furthermore, the multi-variate Boruta approach also showed that some climate datasets, such as the IMERG product in Band-e-Amir, may provide important additional information that may not be included in the reanalysis products. Some datasets were also not considered as important with this method if compared to the correlation analysis, such as the MODIS snow variables in Band-e-Amir. This may be due to multi-collinearity among variables and no new information compared to the better performing reanalysis datasets. Although dataset performance was partly consistent between the regions, especially regarding reanalysis data, the correlation was higher for most variables and more products were found to be suitable for vegetation change analysis in the Band-e-Amir region. The climatic situation of the research areas is one important reason for this difference. The Band-e-Amir region represents a relatively stationary, large scale climate system in Central Asia with seasonally dominating pressure systems^48^. Therefore, anomalies of distant climate stations may be also representative for this region. This situation stands in stark contrast to the Wakhan area which forms the boundary between different climate influence zones, the Westerlies and the Indian summer monsoon^27^. A transition zone leads to a complex situation with spatially and temporarily varying moisture sources and suitable climate products must equally represent both climate systems. This may be impossible for station-based datasets when they are characterized by low gauge density^7,8^. Similarly, satellite data and combined datasets may not be sufficiently calibrated to the regional conditions or to represent both climate systems^112,113^.

The spatial pattern of the correlations also suggests a better representation of precipitation originating from the west, with higher correlation values in Wakhan in the northwest compared to the increasingly monsoonal influenced southeast. However, the majority of the spatial patterns may be explained by small scale differences. In both regions, most non-significant areas were found in valleys and hydrological sinks. The missing correlation in those regions is based on the dominance of other drivers for vegetation growth which are not immediately influenced by climatic variations. Direct human activities, such as agriculture and tillage, which are mostly found in the vicinity of rivers due to the need of irrigation for crops^26,114^, leads to the decoupling of climate and vegetation anomalies cf.^16^. This may be especially true for the Band-e-Amir region and low altitude areas in Wakhan. The separate analysis of different vegetation communities in Wakhan also shows the influence of different ecological drivers. In riparian vegetation communities, precipitation of only one dataset (MERRA-2) was highly suitable for explaining vegetation anomalies and the spatial extent of significant correlations was lower. This indicates that other factors, such as glacial meltwater, flooding or precipitation upstream of the watershed, are also important for variations in vegetation anomalies in those communities or that there are less constraints for vegetation growth due to water limitation in those regions^19^. The better suitability of some variables in alpine grasslands may be explained by the higher importance of snow as water source in high altitudes compared to lower elevations^33^. This is also a reason for the suitability of temperature and the respective negative correlation in this community, as positive temperature anomalies increase direct evaporation of snow and so lead to a reduced moisture availability for vegetation in higher regions.

In conclusion, our research hypotheses on the various datasets were only partly supported by our results and several novel findings contribute to research of global vegetation change. Reanalysis datasets are more appropriate for spatiotemporally consistent and comparable analysis of vegetation changes in regions with poor meteorological infrastructure. The performance of gauge-based datasets, satellite products and combinations of both is regionally variable and some datasets may be problematic for homogenously analyzing conservation or peripheral areas. Snow variables derived from remote sensing sensors with increased resolution were highly suitable but were not better in analyzing vegetation anomalies than several other datasets in this study. Therefore, we suggest the utilization of reanalysis datasets for studying climate-vegetation interrelationships in data poor conservation areas in recent decades. The suitability of respective products also indicates high potential of more complex climate variables that can be derived from reanalysis parameters, such as drought indices^57,81,95^, for upcoming research applications of vegetation anomalies in remote regions.

## Supplementary Information

Supplementary information.

## Data Availability

All utilized raster datasets are available for download free of charge from the respective sources in the reference list.
