# Supplementary material for: Reanalysis datasets outperform other gridded climate products in vegetation change analysis in peripheral conservation areas of Central Asia
Source: Sci Rep. 2020 Dec 31;10:22446. doi: 10.1038/s41598-020-79480-y (PMC7775429; doi:10.1038/s41598-020-79480-y)
Supplement: Supplementary file 1 — Supplementary information. [file 41598_2020_79480_MOESM1_ESM.docx]

# Appendix of ‘Reanalysis datasets outperform other gridded climate products in vegetation change analysis in peripheral conservation areas of Central Asia’

Harald Zandler^1,2*^, Thomas Senftl^1^ and Kim André Vanselow^3^

^1^Working Group of Climatology, Department of Geography, University of Bayreuth, Universitätsstr. 30, 95447 Bayreuth, Germany

^2^Bayreuth Center of Ecology and Environmental Research, University of Bayreuth, Dr. Hans-Frisch-Straße 1-3, 95448 Bayreuth, Germany

^3^ Institute of Geography, Friedrich-Alexander-Universität Erlangen-Nürnberg, Wetterkreuz 15, 91058 Erlangen

*Corresponding author: [harald.zandler@uni-bayreuth.de](mailto:harald.zandler@uni-bayreuth.de)

# Appendix


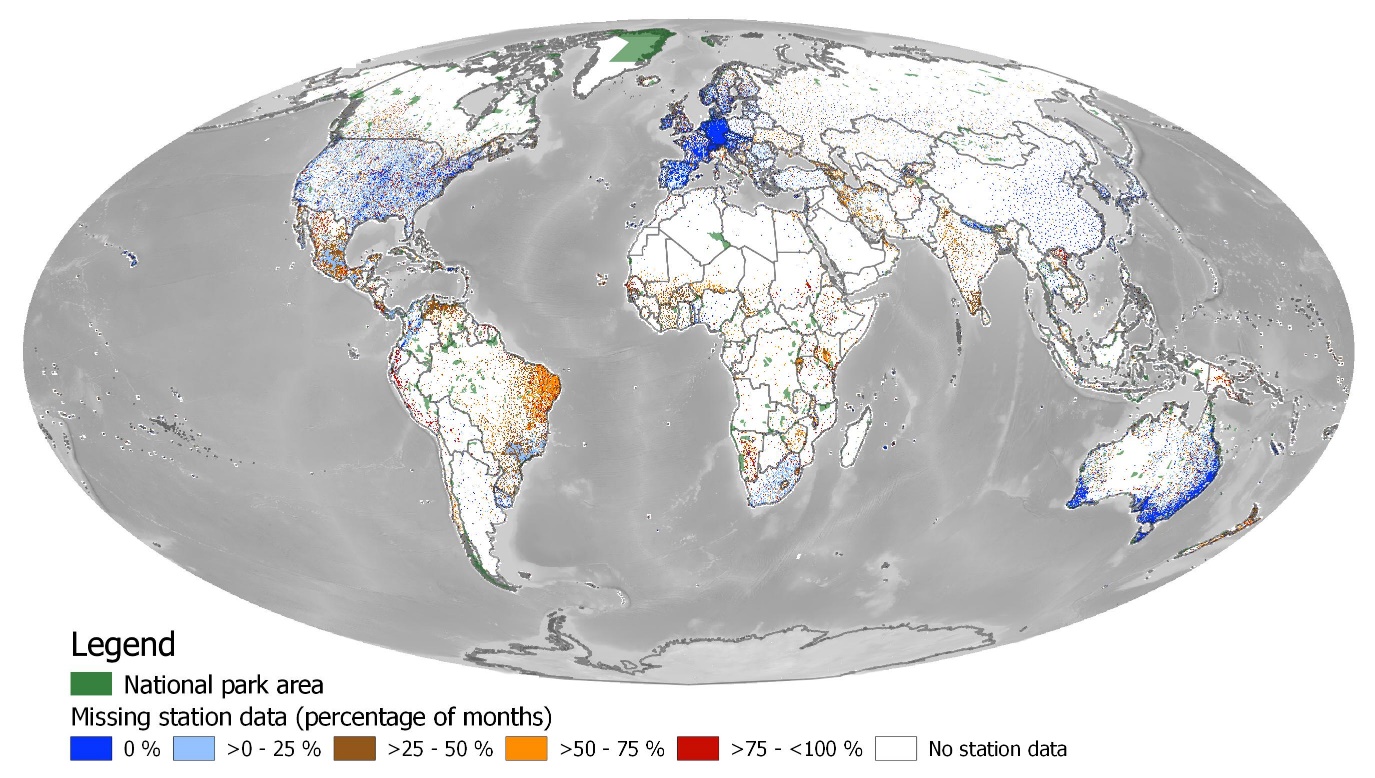


Appendix. 1: Global national park areas and percentage of missing monthly station data input per raster cell in the GPCC Full Data Monthly Product Version 2018 during the climate normal period 1981-2010. Thereby, 91.77% of national park areas are located in cells with no station data during this period. 0.74% are located in cells with > 75 to < 100% of months without station data, 1.29% in cells with > 50 to 75%, 1.58% in cells with > 25 to 50% and 2.65% in cells with > 0 to 25% of months without station data. 1.97% of national park areas are located in cells where all months have a minimum of one station available (National park areas:^4^, GPCC data:^5^, global relief data:^43^, projection: Mollweide). Created using QGIS 3.12 (http://qgis.osgeo.org/)^45^.

Appendix 2. Highly suitable variables (significant values controlled at a FDR < 5%, >=50% of analyzed pixels show significant correlation) and evaluation metrics using the Spearman approach in the Wakhan study region. Descending order based on percentage of significant pixels.

| **Dataset** | **p** | **rho** | **Percentage of significant pixels** |
| --- | --- | --- | --- |
| MERRA-2 precipitation/ Hydrological-year | 0.0000 | 0.84 | 76 |
| MERRA-2 precipitation / Spring-Summer (Mar-Aug) | 0.0000 | 0.83 | 74 |
| MERRA-2 precipitation / Winter half-year (Nov-Apr) | 0.0000 | 0.81 | 71 |
| MERRA-2 precipitation / Spring (Mar-May) | 0.0004 | 0.74 | 70 |
| ERA5-Land soil water / Summer (Jun-Aug) | 0.0056 | 0.62 | 62 |
| CRU precipitation / Spring (Mar-May) | 0.0008 | 0.72 | 60 |
| CRU precipitation / Spring-Summer (Mar-Aug) | 0.0002 | 0.76 | 60 |
| MODIS-NDSI / Spring (Mar-May) | 0.0007 | 0.73 | 59 |
| MODIS-SCD / Spring (Mar-May) | 0.0009 | 0.71 | 58 |
| MODIS-FSC / Spring (Mar-May) | 0.0010 | 0.71 | 58 |
| ERA5-Land precipitation / Spring-Summer (Mar-Aug) | 0.0026 | 0.67 | 57 |
| ERA5-Land skin-reservoir content / Spring-Summer (Mar-Aug) | 0.0043 | 0.64 | 56 |
| MODIS-NDSI / Transition (Feb-Mar) | 0.0023 | 0.67 | 54 |
| MODIS-FSC / Transition (Feb-Mar) | 0.0015 | 0.69 | 50 |

Appendix 3. Highly suitable variables (significant values controlled at a FDR < 5%, >=50% of analyzed pixels show significant correlation) and evaluation metrics using the Spearman approach in the Band-e-Amir study region. Descending order based on percentage of significant pixels.

| **Dataset** | **p** | **rho** | **Percentage of significant pixels** |
| --- | --- | --- | --- |
| ERA5-Land precipitation/ Hydrological-year | 0.0000 | 0.90 | 97 |
| ERA5-Land soil water / Summer (Jun-Aug) | 0.0000 | 0.93 | 96 |
| MERRA-2 precipitation / Spring-Summer (Mar-Aug) | 0.0001 | 0.80 | 95 |
| MERRA-2 precipitation / Winter half-year (Nov-Apr) | 0.0001 | 0.79 | 95 |
| MERRA-2 precipitation/ Hydrological-year | 0.0000 | 0.85 | 95 |
| ERA5-Land soil water / Spring-Summer (Mar-Aug) | 0.0000 | 0.84 | 94 |
| ERA5-Land precipitation / Spring-Summer (Mar-Aug) | 0.0002 | 0.78 | 94 |
| MERRA-2 precipitation / Spring (Mar-May) | 0.0001 | 0.79 | 94 |
| CRU precipitation/ Hydrological-year | 0.0003 | 0.75 | 93 |
| MERRA-2 precipitation / Transition (Feb-Mar) | 0.0008 | 0.72 | 92 |
| MERRA-2 temperature / Spring-Summer (Mar-Aug) | 0.0001 | -0.80 | 92 |
| ERA5-Land temperature / Spring-Summer (Mar-Aug) | 0.0002 | -0.77 | 91 |
| ERA5-Land precipitation / Winter half-year (Nov-Apr) | 0.0006 | 0.73 | 88 |
| MERRA-2 temperature 2m / Spring-Summer (Mar-Aug) | 0.0018 | -0.68 | 86 |
| ERA5-Land skin-reservoir content / Spring-Summer (Mar-Aug) | 0.0000 | 0.86 | 86 |
| IMERG precipitation/ Hydrological-year | 0.0007 | 0.72 | 86 |
| CRU precipitation / Winter half-year (Nov-Apr) | 0.0032 | 0.66 | 85 |
| CRU precipitation / Spring-Summer (Mar-Aug) | 0.0040 | 0.64 | 82 |
| CRU precipitation / Spring (Mar-May) | 0.0038 | 0.64 | 81 |
| ERA5-Land skin-reservoir content/ Hydrological-year | 0.0001 | 0.79 | 80 |
| ERA5-Land skin-reservoir content / Spring (Mar-May) | 0.0000 | 0.83 | 80 |
| IMERG precipitation / Winter half-year (Nov-Apr) | 0.0006 | 0.73 | 80 |
| ERA5-Land precipitation / Spring (Mar-May) | 0.0083 | 0.60 | 79 |
| ERA5-Land soil water/ Hydrological-year | 0.0007 | 0.73 | 79 |
| IMERG precipitation / Spring-Summer (Mar-Aug) | 0.0043 | 0.64 | 78 |
| ERA5-Land skin-reservoir content / Summer (Jun-Aug) | 0.0008 | 0.72 | 78 |
| ERA5-Land temperature/ Hydrological-year | 0.0011 | -0.70 | 77 |
| ERA5-Land snow/ Hydrological-year | 0.0010 | 0.71 | 76 |
| ERA5-Land soil water / Spring (Mar-May) | 0.0029 | 0.66 | 75 |
| MERRA-2 precipitation / Summer (Jun-Aug) | 0.0050 | 0.63 | 75 |
| MERRA-2 temperature / Spring (Mar-May) | 0.0107 | -0.59 | 74 |
| ERA5-Land temperature / Summer (Jun-Aug) | 0.0014 | -0.69 | 73 |
| MERRA-2 temperature 2m / Spring (Mar-May) | 0.0070 | -0.61 | 73 |
| IMERG precipitation / Transition (Feb-Mar) | 0.0063 | 0.62 | 71 |
| ERA5-Land temperature / Spring (Mar-May) | 0.0060 | -0.62 | 70 |
| ERA5-Land precipitation / Summer (Jun-Aug) | 0.0067 | 0.61 | 65 |
| CRU temperature / Spring-Summer (Mar-Aug) | 0.0045 | -0.64 | 64 |
| ERA5-Land snow / Winter half-year (Nov-Apr) | 0.0040 | 0.64 | 63 |
| ERA5-Land snow / Spring-Summer (Mar-Aug) | 0.0018 | 0.68 | 61 |
| MODIS-NDSI / Spring (Mar-May) | 0.0072 | 0.61 | 60 |
| ERA5-Land snow / Spring (Mar-May) | 0.0018 | 0.68 | 59 |
| MODIS-FSC / Spring (Mar-May) | 0.0058 | 0.62 | 59 |
| MERRA-2 temperature 2m/ Hydrological-year | 0.0067 | -0.61 | 58 |
| MODIS-NDSI / Transition (Feb-Mar) | 0.0180 | 0.55 | 55 |
| MODIS-SCD / Spring (Mar-May) | 0.0060 | 0.62 | 55 |
| MODIS-FSC / Transition (Feb-Mar) | 0.0180 | 0.55 | 54 |
| MODIS-NDSI/ Hydrological-year | 0.0042 | 0.66 | 52 |
| ERA5-Land temperature / Spring-Summer (Mar-Aug) two-year average | 0.0076 | -0.62 | 52 |
| MERRA-2 temperature/ Hydrological-year | 0.0156 | -0.56 | 52 |
| ERA5-Land skin-reservoir content / Winter half-year (Nov-Apr) | 0.0022 | 0.67 | 51 |
| MODIS-FSC/ Hydrological-year | 0.0030 | 0.67 | 51 |
| MERRA-2 temperature / Spring-Summer (Mar-Aug) two-year average | 0.0112 | -0.60 | 51 |

Appendix 4. Highly suitable variables (significant values controlled at a FDR < 5%, >=50% of analyzed pixels show significant correlation) and evaluation metrics in the different vegetation communities of the Wakhan region. Descending order based on percentage of significant pixels.

| **Dataset** | **p** | **r** | **Percentage of significant pixels** |
| --- | --- | --- | --- |
| **Riparian vegetation** | | | |
| MERRA-2 precipitation/ Hydrological-year | 0.0001 | 0.80 | 67 |
| MERRA-2 precipitation / Winter half-year (Nov-Apr) | 0.0001 | 0.78 | 64 |
| MERRA-2 precipitation / Spring-Summer (Mar-Aug) | 0.0005 | 0.73 | 60 |
| MERRA-2 precipitation / Spring (Mar-May) | 0.0007 | 0.72 | 59 |
| **Dwarf-shrub cushion steppes** | | | |
| MERRA-2 precipitation/ Hydrological-year | 0.0000 | 0.84 | 79 |
| MERRA-2 precipitation / Winter half-year (Nov-Apr) | 0.0001 | 0.80 | 75 |
| MERRA-2 precipitation / Spring-Summer (Mar-Aug) | 0.0008 | 0.72 | 71 |
| MERRA-2 precipitation / Spring (Mar-May) | 0.0013 | 0.70 | 68 |
| ERA5-Land precipitation / Spring-Summer (Mar-Aug) | 0.0017 | 0.69 | 66 |
| MODIS-NDSI / Spring (Mar-May) | 0.0019 | 0.68 | 62 |
| MODIS-FSC / Spring (Mar-May) | 0.0021 | 0.68 | 62 |
| MODIS-NDSI / Transition (Feb-Mar) | 0.0022 | 0.67 | 61 |
| MODIS-SCD / Spring (Mar-May) | 0.0019 | 0.68 | 61 |
| ERA5-Land soil water / Summer (Jun-Aug) | 0.0013 | 0.70 | 61 |
| MERRA-2 precipitation / Transition (Feb-Mar) | 0.0045 | 0.64 | 59 |
| MODIS-FSC / Transition (Feb-Mar) | 0.0025 | 0.67 | 59 |
| MODIS-SCD / Transition (Feb-Mar) | 0.0024 | 0.67 | 55 |
| ERA5-Land precipitation/ Hydrological-year | 0.0050 | 0.63 | 53 |
| CRU precipitation / Winter half-year (Nov-Apr) | 0.0030 | 0.66 | 52 |
| **Alpine grasslands** | | | |
| MERRA-2 precipitation/ Hydrological-year | 0.0001 | 0.80 | 80 |
| MERRA-2 precipitation / Winter half-year (Nov-Apr) | 0.0002 | 0.77 | 78 |
| MERRA-2 precipitation / Spring-Summer (Mar-Aug) | 0.0010 | 0.71 | 75 |
| MODIS-NDSI / Spring (Mar-May) | 0.0007 | 0.72 | 75 |
| MODIS-FSC / Spring (Mar-May) | 0.0008 | 0.72 | 74 |
| MODIS-SCD / Spring (Mar-May) | 0.0006 | 0.73 | 74 |
| MERRA-2 precipitation / Spring (Mar-May) | 0.0021 | 0.68 | 73 |
| ERA5-Land precipitation / Spring-Summer (Mar-Aug) | 0.0010 | 0.71 | 70 |
| MODIS-SCD / Spring (Mar-May) two-year average | 0.0020 | 0.69 | 65 |
| MODIS-FSC / Spring (Mar-May) two-year average | 0.0025 | 0.68 | 65 |
| ERA5-Land soil water / Summer (Jun-Aug) | 0.0007 | 0.73 | 65 |
| MODIS-NDSI / Spring (Mar-May) two-year average | 0.0031 | 0.67 | 64 |
| ERA5-Land skin-reservoir content / Spring-Summer (Mar-Aug) | 0.0009 | 0.71 | 63 |
| CRU precipitation / Winter half-year (Nov-Apr) | 0.0021 | 0.68 | 63 |
| MODIS-NDSI / Transition (Feb-Mar) | 0.0014 | 0.69 | 62 |
| ERA5-Land snow/ Hydrological-year | 0.0011 | 0.71 | 59 |
| ERA5-Land skin-reservoir content / Summer (Jun-Aug) | 0.0056 | 0.62 | 58 |
| CRU precipitation/ Hydrological-year | 0.0039 | 0.64 | 57 |
| MODIS-FSC / Transition (Feb-Mar) | 0.0020 | 0.68 | 57 |
| CRU temperature / Spring (Mar-May) two-year average | 0.0037 | -0.66 | 56 |
| IMERG precipitation / Spring-Summer (Mar-Aug) | 0.0060 | 0.62 | 56 |
| MERRA-2 precipitation / Transition (Feb-Mar) | 0.0106 | 0.59 | 56 |
| ERA5-Land snow / Spring-Summer (Mar-Aug) | 0.0095 | 0.59 | 55 |
| CRU precipitation / Spring-Summer (Mar-Aug) | 0.0090 | 0.60 | 54 |
| ERA5-Land precipitation / Spring (Mar-May) | 0.0104 | 0.59 | 54 |
| Continued | | | |

Appendix 4: continued

| **Dataset** | **p** | **r** | **Percentage of significant pixels** |
| --- | --- | --- | --- |
| MERRA-2 precipitation / Spring-Summer (Mar-Aug) two-year average | 0.0136 | 0.59 | 54 |
| MODIS-NDSI/ Hydrological-year | 0.0069 | 0.63 | 53 |
| MODIS-FSC / Transition (Feb-Mar) two-year average | 0.0035 | 0.67 | 53 |
| CRU precipitation / Spring (Mar-May) | 0.0087 | 0.60 | 53 |
| MODIS-FSC/ Hydrological-year | 0.0069 | 0.63 | 52 |
| CRU temperature / Spring-Summer (Mar-Aug) two-year average | 0.0068 | -0.63 | 52 |
| MODIS-NDSI / Transition (Feb-Mar) two-year average | 0.0054 | 0.64 | 51 |
